# One-step pretreatment of yellow poplar biomass using peracetic acid to enhance enzymatic digestibility

**DOI:** 10.1038/s41598-017-12542-w

**Published:** 2017-09-22

**Authors:** Hyeong Rae Lee, Romas J. Kazlauskas, Tai Hyun Park

**Affiliations:** 1 School of Chemical and Biological Engineering, Seoul National University, 1 Gwanak-ro, Gwanak-gu, Seoul, 151-744 Republic of Korea; 2 Department of Biochemistry, Molecular Biology & Biophysics and the Biotechnology Institute, University of Minnesota, Saint Paul, MN 55108 USA

## Abstract

Pretreatment of biomass with dilute acid requires high temperatures of >160 °C to remove xylan and does not remove lignin. Here we report that the addition of peracetic acid, a strong oxidant, to mild dilute acid pretreatment reduces the temperature requirement to only 120 °C. Pretreatment of yellow poplar with peracetic acid (300 mM, 2.3 wt%) and dilute sulfuric acid (100 mM, 1.0 wt%) at 120 °C for 5 min removed 85.7% of the xylan and 90.4% of the lignin leaving a solid consisting of 75.6% glucan, 6.0% xylan and 4.7% lignin. Low enzyme loadings of 5 FPU/g glucan and 10 *p*NPGU/g glucan converted this solid to glucose with an 84.0% yield. This amount of glucose was 2.5 times higher than with dilute acid-pretreated solid and 13.8 times higher than with untreated yellow poplar. Thus, the addition of peracetic acid, easily generated from acetic acid and hydrogen peroxide, dramatically increases the effectiveness of dilute acid pretreatment of biomass.

## Introduction

Lignocellulosic biomass is a sustainable, abundant and low-cost resource. Conversion of biomass to biofuels reduces the society’s dependence on petroleum-based fuels and reduces net greenhouse gas emissions^1,2^. Pretreatment of lignocellulosic biomass is key step in the conversion of biomass to biofuels. Lignocellulosic biomass is composed of cellulose, hemicellulose and lignin in an interwoven matrix. Conversion of biomass into biofuels requires enzymatic hydrolysis of cellulose to glucose followed by the microbial fermentation of this glucose to biofuels^3^. The complex structure of biomass makes it recalcitrant to enzymes making the hydrolysis of cellulose slow and inefficient. This recalcitrance is the major economic obstacle to conversion of biomass to sugar^4^. Pretreatment of biomass enhances the accessibility of cellulose to enzymes, thereby overcoming biomass recalcitrance^5^. Effective pretreatments should be energy efficient, not degrade the cellulose and yield a cellulose fraction that is easily hydrolyzed with low enzyme loadings^3,6^.

The most effective pretreatments – dilute acid and organosolv – still require high temperatures of >150 °C. Dilute acid pretreatment at high temperatures (>160 °C) removes most of the hemicellulose with minimal degradation of cellulose and lignin^7,8^. The remaining lignin hinders access of the enzymes to cellulose both by blocking access and by adsorbing the enzymes^9,10^. Organosolv pretreatments using ethanol or THF remove both the hemicellulose and lignin^11–13^, but still require high temperature (>150 °C) and additional steps to recover the organic solvents. Lower temperature pretreatments are desirable because they reduce energy requirements and capital costs of equipment because the pressures generated during pretreatment are lower. In particular, autoclaves conveniently and inexpensively generate temperatures of 121 °C using steam, so pretreatments that work at or below this temperature are desirable.

Peracetic acid is strong oxidant that effectively removes lignin from biomass^14^. Pretreatment with peracetic acid at 80 °C improved the cellulose digestibility of sugarcane bagasse^15^ and pretreatment with hydrogen peroxide-acetic acid mixtures, which generate peracetic acid *in situ*, at 80 °C was also increased the enzymatic digestibility of rice straw, pine wood and oak wood as compared to dilute acid pretreatment^16^. Two-step pretreatment with alkali followed by peracetic acid increased the enzymatic digestibility and reduced the amount of peracetic acid needed^17^. Enhanced enzymatic digestibility of the solid after peracetic acid pretreatment is due to delignification and increased surface area of cellulose^13^. However, pretreatment with only peracetic acid removed mainly lignin leaving most of the hemicellulose. This remaining hemicellulose hindered cellulase access to the cellulose. Removal of both lignin and xylan allows efficient enzymatic hydrolysis of the cellulose^18^.

Yellow poplar (*Liriodendron tulipifera*) grows quickly even in poor soil and sequesters higher amounts of carbon dioxide than other biomass crops due to its extensive root structure. The Korea Forest Service recommended yellow poplar as a biomass crop and it is a major planting species in Korea^19^.

In this study, we increased the effectiveness of dilute acid pretreatment by adding peracetic acid. This single step procedure simultaneously removes both hemicellulose and lignin from yellow poplar biomass under comparably milder conditions. As compared to dilute acid under same conditions, this process reduces pretreatment time, pretreatment temperature and cellulase enzyme loading while providing high yields of glucose.

## Results and Discussion

### Optimization of one-step pretreatment

The four major pretreatment variables are the concentrations of peracetic acid (PAA) and H_2_SO_4_, the temperature and the time of pretreatment. These variables were optimized stepwise for xylan and lignin removal by measuring the effect of one variable, while the other variables were fixed at the harsh condition.

**Figure 1:**
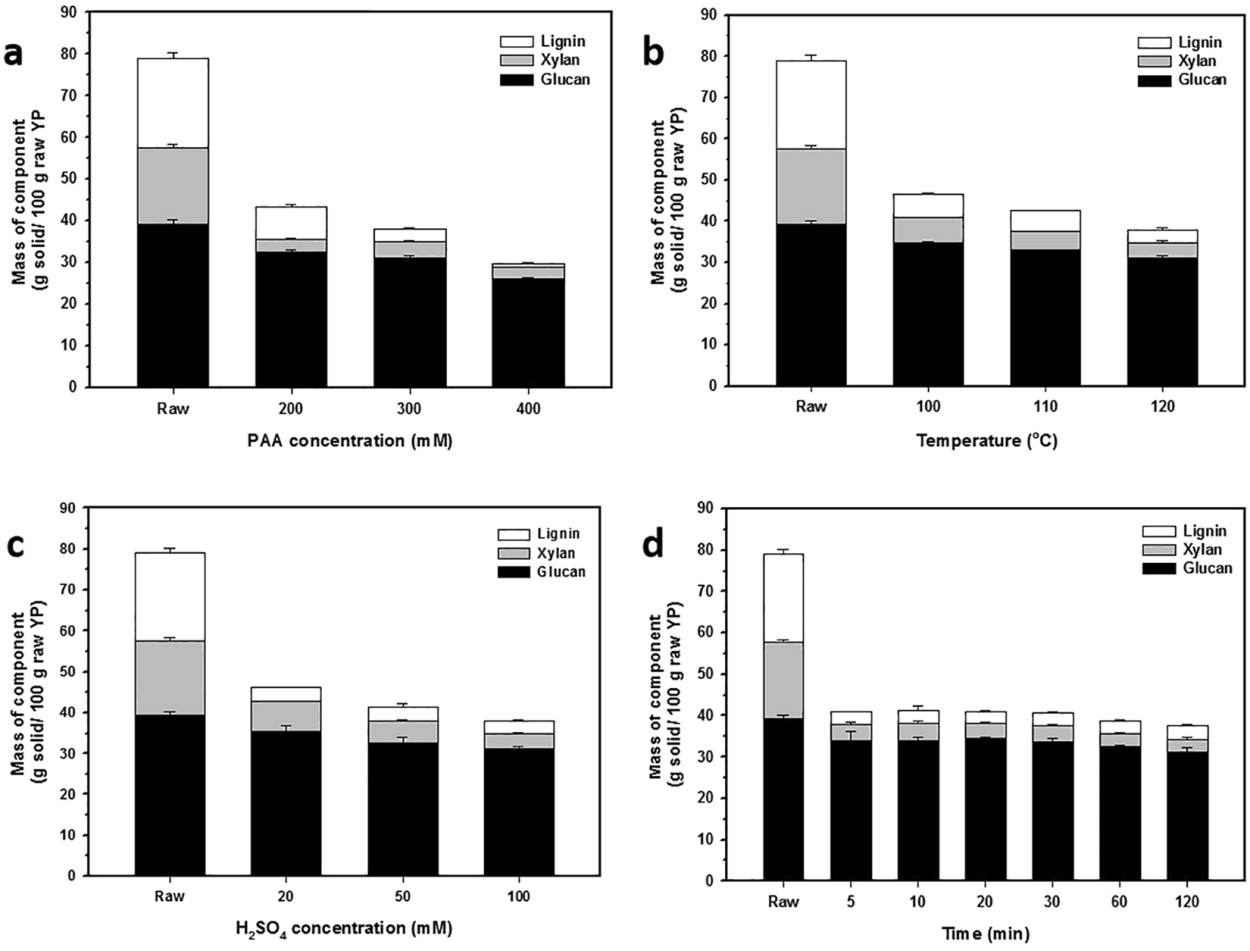
Optimization of one-step pretreatment. Increasing PAA concentration, pretreatment temperature and H_2_SO_4_ concentration increased xylan and lignin removal. The pretreatment time of 5 min was sufficient for the removal of xylan and lignin. Composition analysis of pretreated solid with (**a**) different PAA concentrations at 120 °C with 100 mM H_2_SO_4_ for 2 h, (**b**) different pretreatment temperatures with 300 mM PAA and 100 mM H_2_SO_4_ for 2 h, (**c**) different H_2_SO_4_ concentrations with 300 mM PAA at 120 °C for 2 h, (**d**) different pretreatment times with 300 mM PAA and 100 mM H_2_SO_4_ at 120 °C. Error bars correspond to the standard deviation for three measurements.

A PAA concentration of 300 mM gave the highest xylan and lignin removal with minimal loss of glucan. The ratio of PAA solution to water was varied from 1:9, 2:8 and 3:7 (v/v), which corresponded to 200, 300 and 400 mM PAA, respectively. Caution: PAA decomposes at high temperatures. Solutions with >600 mM PAA rapidly evolved gas over 100 °C so were not used. The other pretreatment conditions were 120 °C, 100 mM H_2_SO_4_ and 2 h pretreatment time. As the concentration of PAA increased, the amount of lignin removed increased from 63.1% in 200 mM, 85.4% in 300 mM, to 96.2% in 400 mM, but the amount of xylan removed remained similar (84.1, 79.4, 85.2%, respectively) (Fig. 1a). In the pretreatment, PAA reacted with the lignin^14^, while the acidic conditions were responsible for xylan hydrolysis. With increasing PAA concentration, the losses of glucan also increased to 17.0, 20.8 and 33.6%, respectively. The optimal PAA concentration was 300 mM because it gave a high xylan and lignin removal and acceptable loss of glucan.

The optimal temperature for removal of xylan and lignin was 120 °C (Fig. 1b). Pretreatment at 100 or 110 °C with 300 mM PAA for 2 h only partially removed xylan and lignin, but pretreatment at 120 °C effectively removed both.

The higher acid concentration of 100 mM removed xylan more effectively, but had no effect on lignin removal (Fig. 1c). High acid concentration (100 mM H_2_SO_4_), removed more xylan (79.4%) than at the low acid concentrations (25 mM H_2_SO_4_: 60.7%, 50 mM H_2_SO_4_: 71.0%), indicating that H_2_SO_4_ catalyzes xylan hydrolysis. When combined with PAA, more effective xylan removal leads to more effective lignin removal. PAA with added H_2_SO_4_ removed lignin more effectively than PAA only^20^, but the amount of lignin removal did not correlate with increasing concentration of H_2_SO_4_ at 120 °C. Therefore, optimal concentration of 100 mM H_2_SO_4_ was selected to optimize the xylan hydrolysis.

Surprisingly, pretreating for only 5 min was as effective as pretreating for 2 h (Fig. 1d). Heating the sample to 120 °C and cooling required approximately 3 min, so it was not practical to reduce the pretreatment time below 5 min. Xylan and lignin removal were complete within 5 min and extending the pretreatment time had little further effect on xylan hydrolysis or delignification. Separate experiments revealed that the concentration of PAA decreased from 300 mM to ~30 mM after 5 min at the pretreatment conditions. Longer pretreatment times also had the disadvantage of removing some of the glucan due to acid hydrolysis. For example, ~12, ~15 and ~20% of glucan was lost after a 5, 30 and 120-min pretreatment (Fig. 1d).

**Figure 2:**
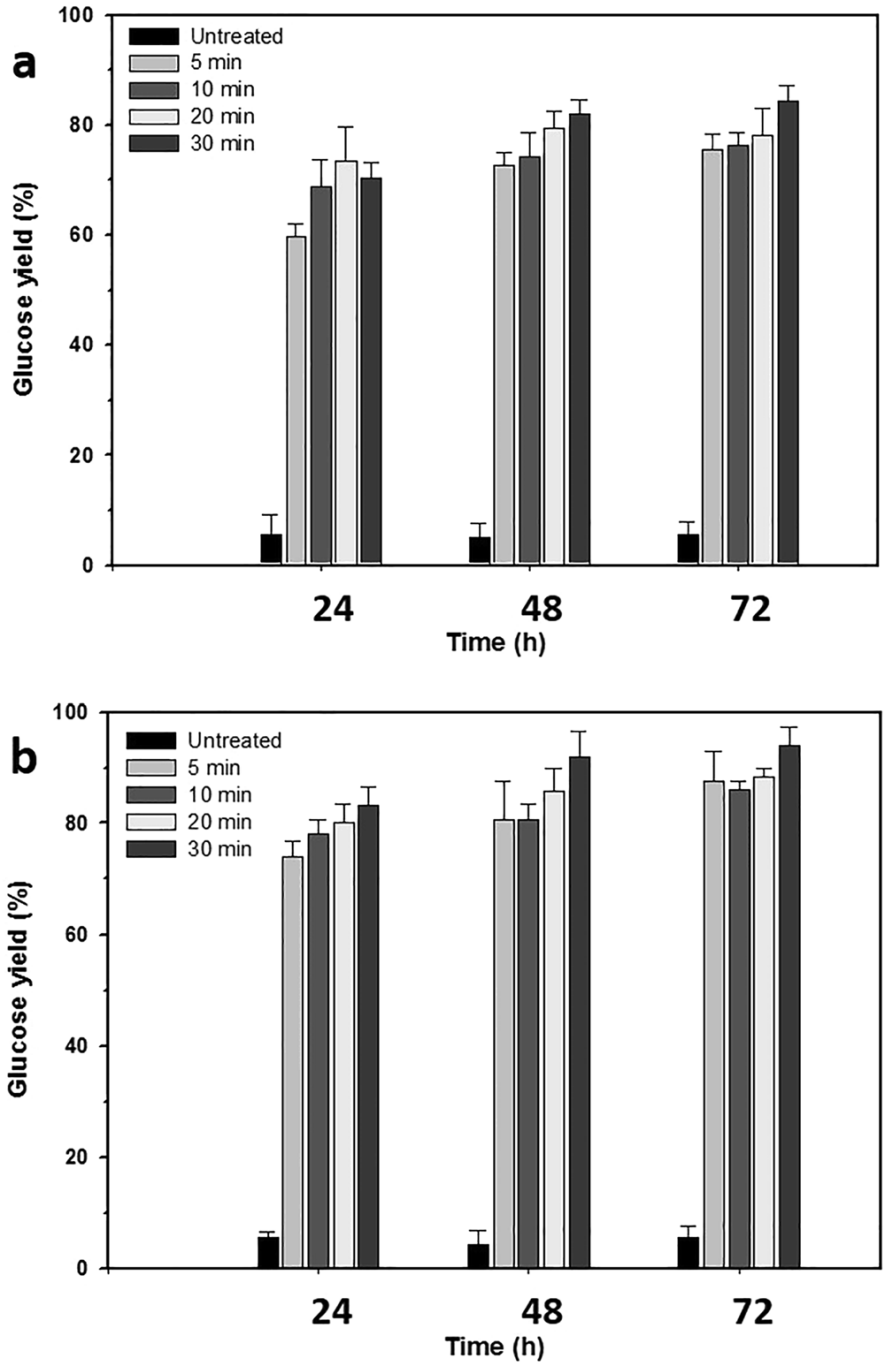
Effect of pretreatment time on enzymatic digestibilities of pretreated solid. The solid pretreated for 5 min released slightly lower amounts of glucose than the solids pretreated for 10, 20 and 30 min. Yellow poplar was pretreated with 300 mM PAA and 100 mM H_2_SO_4_ at 120 °C. Enzymatic hydrolysis was conducted using enzyme loadings of (**a**) 5 FPU/g glucan and (**b**) 30 FPU/g glucan. Both solutions contained 30 *p*NPGU/g glucan. Error bars correspond to the standard deviation for three measurements.

The enzymatic digestibilities of the solids from different pretreatment times were similar (Fig. 2). At an enzyme loading of 5 FPU/g glucan and 72 h digestion, the glucose yield increased slightly with increasing pretreatment time (75.5, 76.2, 78.1 and 84.3%). Samples pretreated for 30 min yielded ~10% more glucose than those pretreated for only 5 min. At the higher enzyme loading of 30 FPU/g glucan, the glucose yield increased and the differences between the different pretreatment times decreased: 87.5, 86.1, 88.2 and 93.9%. These similar glucose yields from 5, 10 and 20 min pretreated solids suggests that the pretreatment removed similar amounts of xylan and lignin. The 10% higher glucose yield after 30 min pretreatment comes at the energy cost of longer pretreatment. We selected 5 min as the optimal pretreatment time, but others could choose a longer pretreatment to increase the glucose yield.

Negligible amounts of sugar degradation products, furfural or HMF, formed under the selected pretreatment conditions. These degradation products, which can also inhibit subsequent fermentation, often form under hasher conditions^10^.

Finally, optimal conditions were selected to minimize loss of glucan and formation of byproducts, and enhance xylan and lignin removal. Therefore, the optimal conditions for one-step pretreatment were 300 mM PAA (~2.3 wt%), 100 mM H_2_SO_4_ (~1 wt%), 120 °C and 5 min. These optimal conditions are milder than those for the standard acid hydrolysis of biomass (4 wt% sulfuric acid at 120 °C for 1 h)^21^.

### Comparison with dilute acid pretreatment under same conditions

The effectiveness of the one-step pretreatment was compared to the standard dilute acid pretreatment under the same conditions of 120 °C for 5 min.

### Chemical composition

Raw yellow poplar biomass contains 39.3 g glucan, 18.4 g xylan, 21.4 g lignin and 20.9 g other components per 100 g. Dilute acid (DA) pretreatment selectively solubilized and hydrolyzed xylan, thereby increasing the relative amounts of glucan and lignin in the remaining solid. The DA-pretreated solid contained 38.2 g glucan, 4.4 g xylan and 17.0 g lignin per 100 g of raw YP, which corresponds to removal of 75.8% of the original xylan and 20.8% of the lignin. DA pretreatment at higher temperatures removes 90 to 100% of the xylan^3,13^, but at the moderate temperature of 120 °C DA pretreatment removed only 75.8% of the xylan.

**Figure 3:**
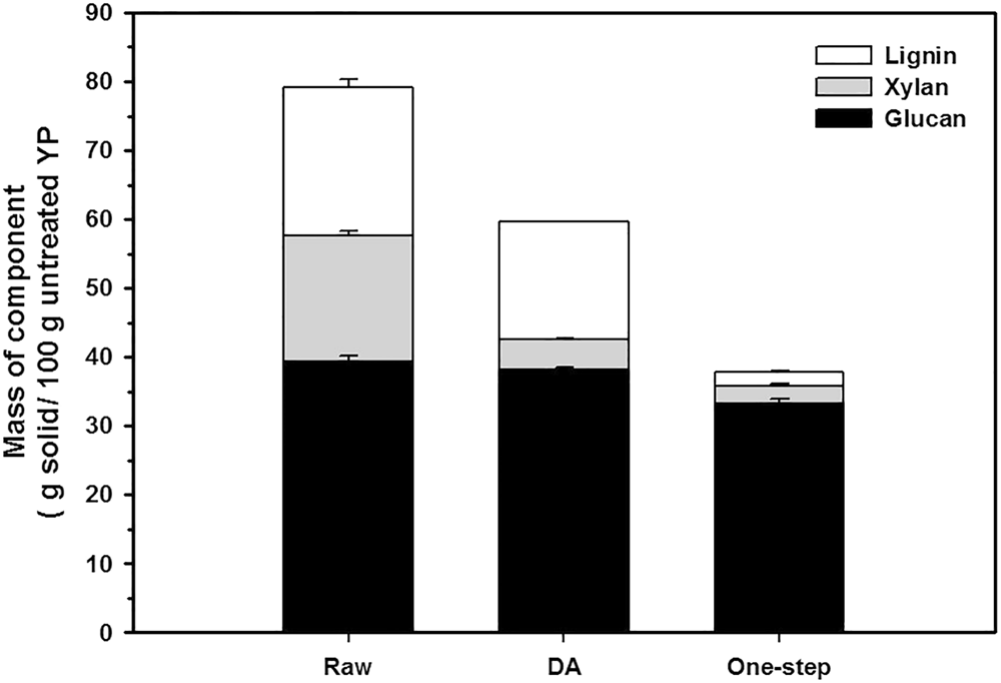
Composition of pretreated solid by DA and one-step DA/PAA pretreatment under same conditions. While DA pretreatment mainly hydrolyzed the xylan, one-step pretreatment solubilized both xylan and lignin. The values are based on the content of each component in 100 g of yellow poplar before pretreatment. Pretreatment conditions: DA: 100 mM H_2_SO_4_, 120 °C, 5 min; One-step: 300 mM PAA, 100 mM H_2_SO_4_, 120 °C, 5 min. Error bars correspond to the standard deviation for three measurements.

The DA/PAA pretreatment removed both xylan and lignin leaving a glucan-rich solid (Fig. 3). The DA/PAA-pretreated solid contained 33.2 g glucan, 2.6 g xylan and 2.1 g lignin per 100 g of raw YP, which corresponds to removal of 85% of the original xylan and 90% of the lignin under moderate temperature. The relative amount of glucan in the solid fraction increased from 39.3% in the raw biomass to 75.6% in the pretreated biomass. Only 15.4% of the glucan was lost during this one-step pretreatment. The one-step DA/PAA pretreatment dramatically improved the lignin removal and also increased the xylan removal as compared to DA pretreatment under same conditions.

### Enzymatic hydrolysis

**Figure 4:**
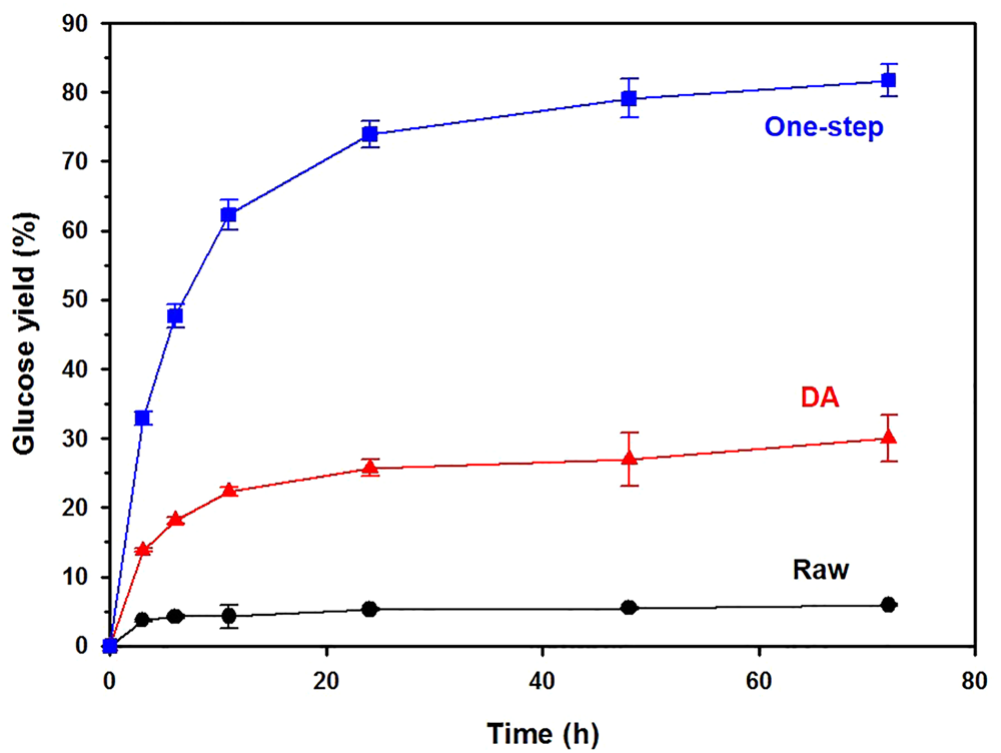
Comparison of glucose yield from enzymatic hydrolysis of solid from DA and one-step pretreatment of yellow poplar using 5 FPU/g glucan and 10 *p*NPGU/g glcuan. Glucose yield of raw YP and DA-pretreated solid was 5.9 and 30.0%, but increased up to 81.7% with one-step pretreatment at 72 h. Delignification in biomass increased the accessibility of cellulase to cellulose and reduced the inhibitory effect of lignin to cellulase compared as DA pretreatment.

Cellulase mixtures digested the one-step pretreated solid more effectively than the raw YP or the DA-pretreated solid (Fig. 4). The enzymatic hydrolysis released only 5.9 and 30.0% of the glucose in raw YP and DA-pretreated solids, respectively, but released 81.7% of the glucose from the one-step pretreated solid after 72 h at low enzyme loadings. The one-step pretreated solid had 13.8 and 2.5 times higher enzymatic digestibility than raw YP and DA-pretreated solid, respectively. In the DA-pretreated solid, the residual lignin or pseudo-lignin (re-deposited lignin) during DA pretreatment likely blocked access of the enzymes to cellulose and non-productively adsorbed the cellulases thereby preventing them from acting on the cellulose^22,23^. The higher rate of removal of xylan and especially lignin in the one-step pretreated biomass enhanced its enzymatic digestibility by increasing the accessibility of cellulase to cellulose and by reducing inhibition by residual lignin, thereby reducing the enzyme requirements.

The optimal conditions for one-step pretreatment (120 °C, 5 min) are milder than those for various organosolv pretreatments, Table 1. Organosolv pretreatment use organic solvents and catalysts to simultaneously remove xylan and lignin from lignocellulosic biomass resulting in a cellulose-rich fraction. To remove comparable amounts of xylan and lignin, organosolv pretreatment required substantial amounts of organic solvent and higher temperatures or longer times than the one-step pretreatment. In addition, pretreatment with PAA did not require subsequent incubation to enhance the cellulose digestibility as compared to pretreatment with formic acid or γ-valerolactone.

**Table 1:** Organosolv biomass pretreatment for saccharification.

Biomass	Solvent	Catalyst	Temp. (°C)	Time (min)	Cellulose yield (%)	Hemicellulose removal (%)	Delignification (%)	Cellulose digestibility (%)	Ref.
Lodgepole pine	Ethanol 65% (v/v)	1.1% H_2_SO_4_	170	60	74.8	93.0	69.5	~100	29
Wheat straw	Ethanol 60% (w/w)	0.29% H_2_SO_4_	190	60	91.1	95.3	75.8	89.4	30
Sugarcane bagasse	Glycerol 80% (w/w)	0.94% H_2_SO_4_	190	60	89.3	96.6	53.5	>90	31
Sugarcane bagasse	FA^a^ 88% (w/w)	—	107	60	87.5	90.7	74.5	53.2 (95.7^b^)	32
Sugarcane bagasse	PAA^c^ 50% (on biomass)	—	80	120	—	—	82.0	82.1	33
Parairie cordgrass	MIBK^d^ 9% (w/w)	0.69% H_2_SO_4_	154	39	—	—	87.0	84.0	34
Switchgrass	EA^e^37%, ethanol 25% (w/w)	0.46% H_2_SO_4_	140	20	—	—	—	84.9	35
Beech wood	2-MTHF^f^ 50% (v/v)	0.1 M oxalic acid	140	180	—	—	—	—	36
Corn stover	THF^g^ 50% (v/v)	0.5% H_2_SO_4_	150	25	75.0	94.8	76.6	95.0	11
Beech wood	GVL^h^ 80% (w/w)	0.75% H_2_SO_4_	120	60	95.0	78.5	77.0	55.0 (99.0^i^)	37
Yellow poplar	PAA 2.3% (w/v)	1% H_2_SO_4_	120	5	75.6	85.0	90.0	81.7	This study

^a^Formic acid. ^b^Deformylation with NaOH incubation at 120 °C for 1 h. ^c^Peracetic acid. ^d^Methyl isobutyl ketone. ^e^Ethyl acetate. ^f^2-Methyltetrahydrofuran. ^g^Tetrahydrofuran. ^h^γ-Valerolactone. ^i^NaOH incubation at 50 °C for 1 h and neutralization with acetic acid.

The one-step pretreatment yields a lower quality xylose fraction than DA pretreatment. The xylose fraction is a mixture of xylose, xylose degradation products, lignin and acetic acid, while the DA pretreatment yields mainly an acidic xylose fraction. In addition, HPLC analysis revealed three sugar oxidation products, likely due to reaction with the PAA. These products were not further characterized. These products could also be fermentation inhibitors, but HMF, furfural and these products were removed in the washing step before enzymatic hydrolysis. Recently engineered microbes can convert not only glucose, but also xylose and arabinose, to fuels. The loss of the xylose/arabinose fraction is a disadvantage of the one-step pretreatment, which must be balanced by the short pretreatment time, higher quality of the glucose fraction, and high yield of glucose at low enzyme loadings.

### Structural characterization

The structural changes in crystallinity, functional group distribution and surface morphology are consistent with removal of xylan and lignin using the one-step pretreatment.

**Figure 5:**
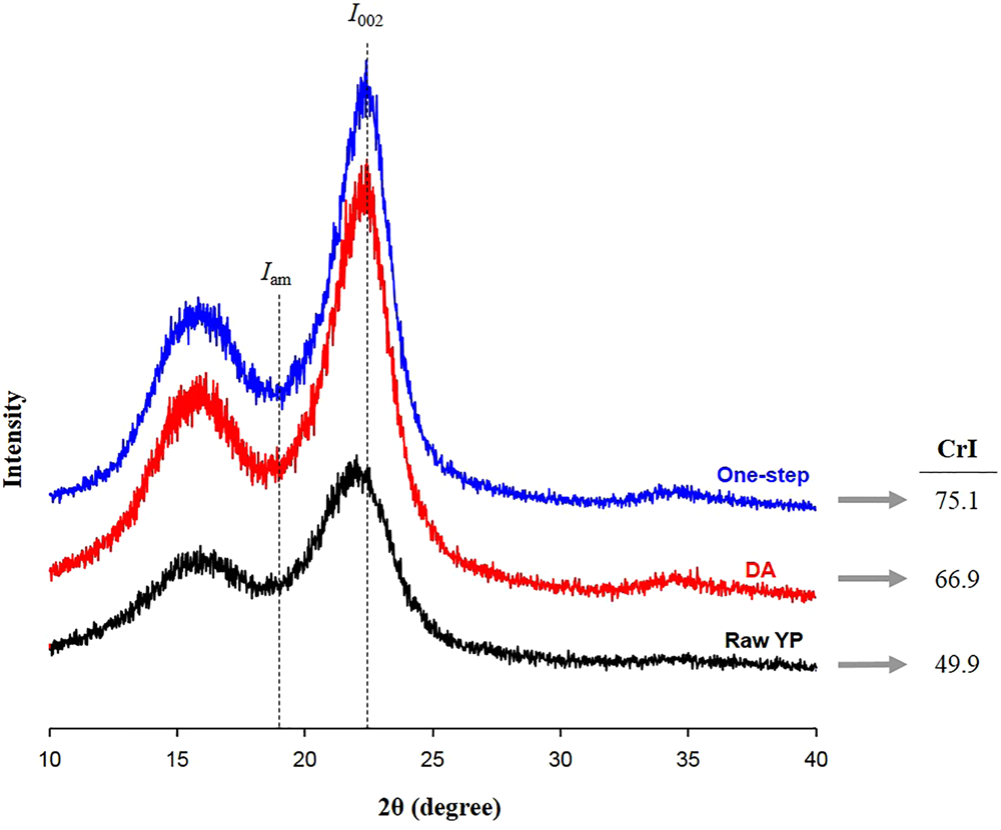
Crystallinity changes after DA pretreatment and one-step pretreatment. The crystallinity of one-step pretreated solid increased due to high removal of xylan and lignin. I_002_: maximum intensity of crystalline portion at ~22° and I_am_: minimum intensity of amorphous portion at ~18°.

The crystallinity index (CrI) of one-step pretreated solid increased from 49.9% for raw YP to 66.9% for DA-pretreated solid and to 75.1% for the one-step pretreated solid (Fig. 5). The CrI indicates the relative amount of crystalline cellulose in the solid. This increase is consistent with the removal of the amorphous xylan and lignin fractions during pretreatment.

**Figure 6:**
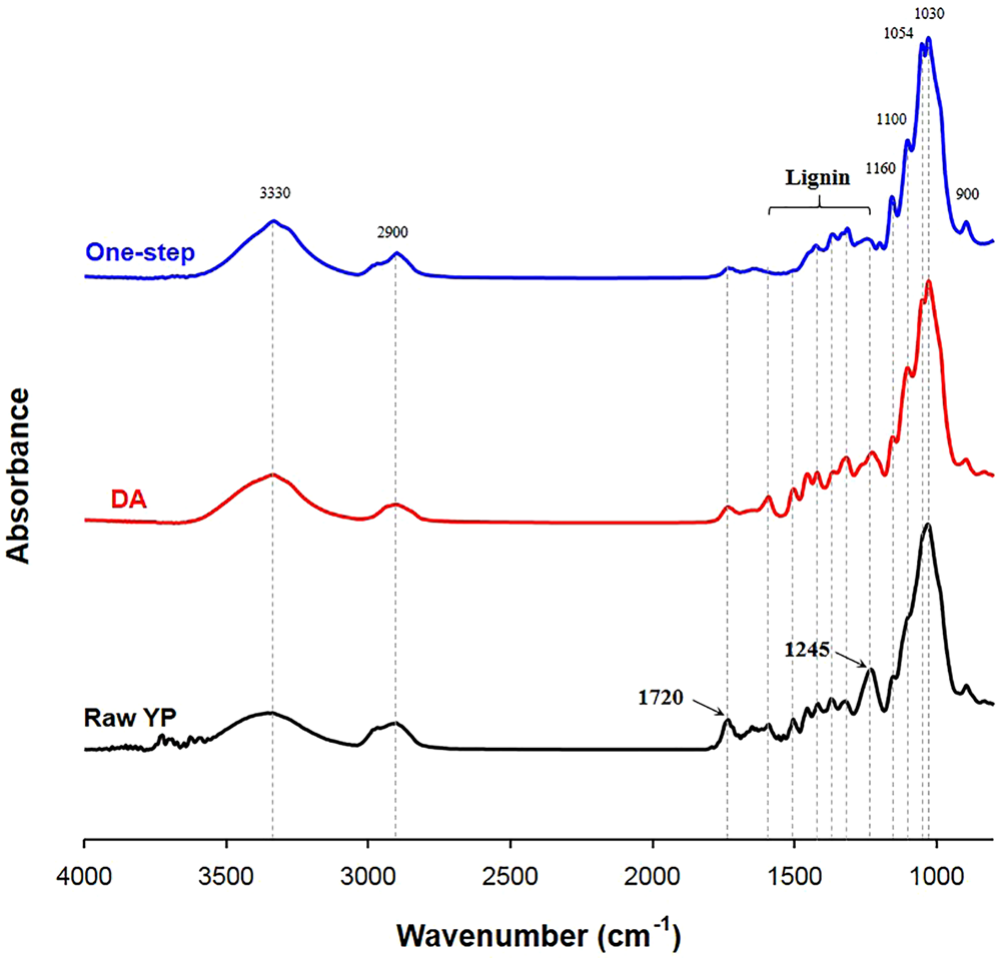
FT-IR spectra of raw yellow poplar, DA-pretreated solid and one-step pretreated solid. After one-step pretreatment, the absorption band related hemicellulose and lignin decreased, and the band associated with cellulose increased.

The absorption bands in the FTIR spectrum related to functional groups in hemicellulose and lignin decreased in the one-step pretreated solid as compared to that of raw YP (Fig. 6). The ester linkage C = O between lignin and hemicellulose at 1720 cm^−1^ and the C-O-C stretch of the acetyl group in hemicellulose at 1245 cm^−1^ disappeared. The absorption bands at 1300–1600 cm^−1^ related to lignin also decreased. These decreases indicate that the one-step pretreatment removed xylan and lignin. In addition, several absorption bands associated with cellulose increased in the one-step pretreated solid as compared to that of raw YP. The O-H stretch at 3330 cm^−1^ and C-H stretch at 2900 cm^−1^ associated with cellulose were stronger after one-step pretreatment than raw YP. Similarly, the C-O-C glycosidic bond stretching at 1160 cm^−1^, C-O-C ring skeletal vibration at 1100 cm^−1^ and C-O-H stretching of primary and secondary alcohols at 1030 cm^−1^ were also more intense than in raw YP. These increases are consistent with an increase in the relative amount of glucan. In contrast, after DA pretreatment, only the absorption bands related to hemicellulose decreased, while the bands related to lignin increased. This difference is consistent with selective removal of xylan by the DA pretreatment, leaving a glucan- and lignin-enriched solid.

**Figure 7:**
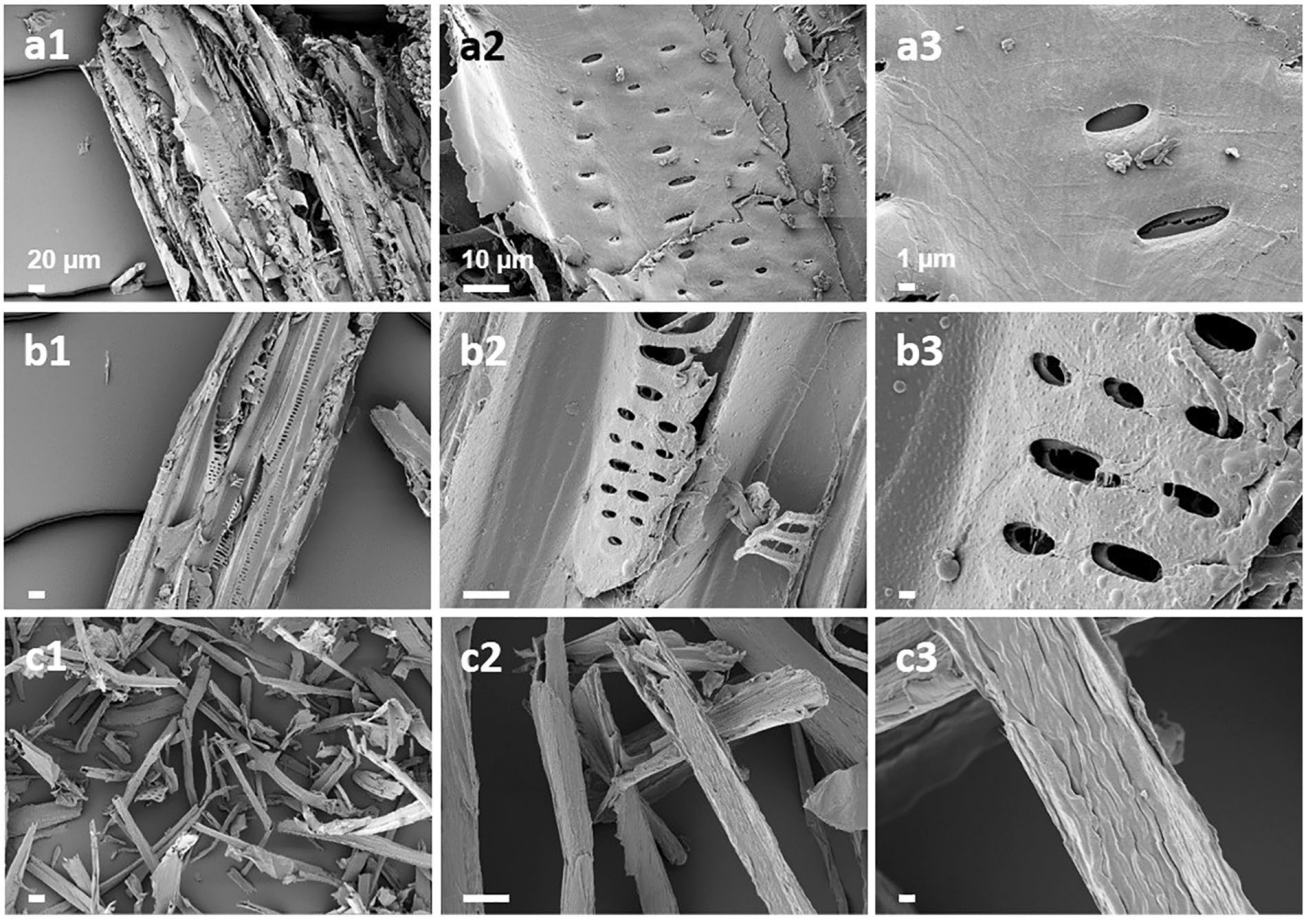
SEM images of raw yellow poplar (**a**), DA-pretreated solid (**b**), and one-step pretreated solid (**c**) at magnification 500 × (1), 3,000 × (2), and 10,000 × (3). Scale bars are shown. The raw YP had a rigid and compact surface. DA-pretreated solid showed enlarged pores and surface covered by a thin layer of deposited lignin. One-step pretreated solid showed completely defibrated structure and smooth cellulose fiber.

The surface morphology of the solid after one-step pretreatment showed an extensively disrupted structure with exposed cellulose fibers (Fig. 7). The surface of raw YP appeared compact with rigid and highly ordered fibrils, and some flakes on the surface (Fig. 7a). The surface of the solid pretreated with DA showed enlarged pores and irregular cracks, but preserved the major features of raw YP. A thin layer covered the surface, likely consisting of redeposited lignin, which can inhibit binding of the cellulase to cellulose^24^. The surface of the one-step pretreated solid showed completely different surface morphology (Fig. 7c). The structure separated into fibers with the width of typical fragment decreasing from >250 µm in raw YP to ~10 µm. Similar decreases in the width of fibers in sugar cane bagasse occurred upon pretreatment with concentrated acetic acid and hydrogen peroxide^25^. In addition, the pores disappeared and the surface appeared smooth, which is consistent with the removal of xylan and lignin. One-step pretreatment destroyed the structure of YP and exposed the cellulose fibers, thereby increasing accessibility of cellulase to cellulose.

## Conclusions

A short (5 min) one-step pretreatment at 120 °C with 300 mM PAA and 100 mM H_2_SO_4_ removed both xylan and lignin from yellow poplar biomass in contrast to dilute acid pretreatment, which removed mainly xylan under same conditions. Enzymatic hydrolysis of solid from this one-step pretreatment released ~80% of the glucose, which was 2.5 and 13.8 times higher than from solids from DA-pretreatment and raw YP, respectively. The difference between one-step and DA pretreatment was high lignin removal, which dramatically enhanced enzymatic hydrolysis at low enzyme loadings. In addition, one-step pretreatment decreased the formation of fermentation inhibitors. This novel one-step pretreatment under mild conditions is a promising method for more efficient in biofuel production.

## Methods

### Materials

20-year-old yellow poplar (*Liriodendron tulipifera*) was purchased from NutraPharm Tech (Korea) and all bark, branches and leaves were removed. The wood was milled to small particles using a grinder (MHK Trading Company, Korea). The particles were sieved through a 40–60 mesh (0.25 ~ 0.42 mm hole), air dried at 50 °C and stored in a desiccator at room temperature until use.

### One-step pretreatment of yellow poplar biomass

Peracetic acid was prepared by adding sulfuric acid (50 or 100 mM) to a 1:1 (v/v) mixture of 30% hydrogen peroxide and acetic acid and allowing the mixture to equilibrate at room temperature for 72 h. The concentration of peracetic acid was measured using the methyl tolyl sulfide assay^26^.

Yellow poplar (YP) wood particles (0.2 g) were loaded into a 10-mL heavy-walled Pyrex® glass tube followed by 4 mL of solution containing 1:9, 2:8 or 3:7 volume ratio of peracetic acid solution from above to water. Depending on the experiment, sulfuric acid was added to form a 25, 50 or 100 mM sulfuric acid solution. The glass tube was sealed by PTFE-silicon septa with snap-top cap and put into a microwave reactor (CEM Discover; CEM Corporation, USA) at the desired temperature for the designated residence time. The time was counted from the time that the reaction mixture reached the desired temperature, which was within 3 min of the start. After pretreatment, the glass tube was removed from the reactor and cooled within 1 min by immersing in water. The slurry was filtered through a P4 glass filter crucible (DURAN, Germany) and the residue was washed extensively with water. After washing, the residue was stored at 4 °C until enzymatic hydrolysis. A sample of this residue was dried in an oven at 105 °C for compositional analysis and characterization. HPLC analysis of the filtrate determined the amount of solubilized sugars and degraded compounds.

### Enzymatic hydrolysis of pretreated yellow poplar

Enzymatic hydrolysis of raw YP and pretreated solids was conducted in triplicate on a shaking incubator at 150 rpm and 50 °C following NREL standard protocols^27^. A substrate amount equivalent to 0.1 g glucan (based on dry weight) was loaded into a 15 mL glass vial. Sodium azide solution (100 µL of 20 mg/mL) was added as an antimicrobial. Citrate buffer (50 mM, pH 4.8) was added to make the final liquid volume of 10 mL. The slurry was put into the incubator at 50 °C for 1 h before addition of enzyme. Cellulase (Celluclast 1.5 L, Sigma, USA, 5 or 30 FPU/ g glucan) and β–glucosidase (Novozyme 188, Sigma, 10 or 30 *p*NPGU/ g glucan) were loaded into each glass vial. The activities of cellulase and β–glucosidase were 319 FPU/mL and 118 *p*NPGU/mL, respectively. Aliquots (200 µL) were removed at specific intervals for sugar yield determination by HPLC. The glucose yield (%) was calculated from the amount of glucose generated by enzymatic hydrolysis divided by amount of glucan in each pretreated solid, adjusted for the weight increase upon hydrolysis, and multiplied by 100%.

## Analytical Methods

### Chemical composition analysis

The amount of acid-insoluble lignin was measured according to NREL standard protocol^21^. Raw YP wood, DA-pretreated or one-step pretreated solid (0.3 g) was suspended in 3 mL of 72 wt% sulfuric acid. The slurry was stirred every 15 min to disperse the material. After 1 h, the slurry was diluted with 84 mL of water to form 4 wt% sulfuric acid. The slurry was autoclaved at 121 °C for 1 h, then cooled to room temperature and filtered through P4 glass filter crucibles (DURAN). The filtrate was used for sugar analysis and the washed residue was dried at 105 °C to determine acid-insoluble lignin. All the samples were measured in triplicate.

### Analysis of sugars and degradation compounds

Sugars and degradation compounds in filtrate were measured using an HPLC (UltiMate 3000, Thermo Fisher Scientific, USA) equipped with a refractive index detector and an Aminex HPX-87H column (300 × 7.8 mm, 5 µm; Bio-Rad, USA) eluted with 5 mM sulfuric acid at a flow rate of 0.6 mL/min at 60 °C. Authentic glucose, xylose, acetic acid, furfural and HMF showed retention times of 8.7, 9.4, 14.3, 27.6 and 40.9 min, respectively.

### Crystallinity

The crystallinity of the solids was analyzed using a powder X-ray diffractometer (D8 Advance, Bruker, Germany). The solids were scanned within 5–40° at 0.04 °/s. The crystallinity index (CrI) was calculated according to^28^:

1 $$CrI( \% )=\frac{{I}_{002}-{I}_{am}}{{I}_{am}}\times 100$$

where I_002_ is the maximum intensity of crystalline portion at ~22° and I_am_ is the minimum intensity of amorphous portion at ~18°.

### Fourier transform infrared (FTIR) spectroscopy

Chemical functional groups in the solids were characterized by FTIR (Nicolet 6700, Thermo Scientific, USA) using the attenuated total reflectance accessory. The spectra were acquired in a range from 4000 to 650 cm^−1^ by 32 scans with a resolution of 8 cm^−1^.

### Field emission-scanning electron microscopy (FE-SEM)

The surface morphology of the solids was examined using a field-emission scanning electron microscope (FESEM; AURIGA, Carl Zeiss, Germany) at an acceleration voltage of 2 kV. Before observation, air-dried samples of each were placed on a stub with carbon tape and sputter-coated with platinum.
